# Water droplet can mitigate dust from hydrophobized micro-post array surfaces

**DOI:** 10.1038/s41598-021-97847-7

**Published:** 2021-09-15

**Authors:** Abba Abdulhamid Abubakar, Bekir Sami Yilbas, Al-Qahtani Hussain, Ghassan Hassan, Johnny Ebaika Adukwu

**Affiliations:** 1 Mechanical Engineering Department, KFUPM, Dhahran, 31261 Saudi Arabia; 2 Interdisciplinary Research Center in Renewable Energy and Power, Mechanical Engineering Department, KFUPM, Dhahran, 31261 Saudi Arabia; 3 K.A. CARE Energy Research & Innovation Center at Dhahran, Dhahran, Saudi Arabia

**Keywords:** Mechanical engineering, Colloids, Fluids

## Abstract

Water droplet rolling motion over the hydrophobized and optically transparent micro-post array surfaces is examined towards dust removal pertinent to self-cleaning applications. Micro-post arrays are replicated over the optically transparent polydimethylsiloxane (PDMS) surfaces. The influence of micro-post array spacing on droplet rolling dynamics is explored for clean and dusty surfaces. The droplet motions over clean and dusty micro-post array surfaces are monitored and quantified. Flow inside the rolling droplet is simulated adopting the experimental conditions. Findings reveal that micro-post gap spacing significantly influences droplet velocity on clean and dusty hydrophobized surfaces. Air trapped within the micro-post gaps acts like a cushion reducing the three-phase contact line and interfacial contact area of the rolling droplet. This gives rise to increased droplet velocity over the micro-post array surface. Droplet kinetic energy dissipation remains large for plain and micro-post arrays with small gap spacings. A Rolling droplet can pick up dust particles from micro-post array gaps; however, few dust residues are observed for large gap spacings. Nevertheless, dust residues are small in quantity over hydrophobized micro-post array surfaces.

## Introduction

The hydrophobic wetting state of surfaces finds a wide range of applications in many fields including energy harvesting^1^, biomedical technology and medicine^2^, self-cleaning^3^, and similar. Hydrophobic state demonstrates water-repellant characteristics over the surface, which becomes critical for water droplet motion in terms of rolling and sliding. Because of the topology of surface texture, air-trap in between the texture gaps enables reducing three-phase contact line (water–air-surface). This lowers the lateral surface tension force component at the droplet liquid–solid interface while easing interfacial retarding and pinning forces acting over the liquid droplet. The spacing between the pillars in the surface texture morphology becomes important for achieving minimum interfacial resistance during droplet rolling. Increasing pillar spacing can cause droplet fluid reaching the pillar bottoms and the droplet fluid can wet the surface. This changes the wetting state from Cassie & Baxter to Wenzel. Similarly, the hierarchal distribution of texture pillars becomes essential in maintaining the Cassie & Baxter state over the entire surface. The hierarchical distribution of pillars can be rearranged to create sufficiently spaced micro-posts while creating micro-post-arrays over the surface. Although water repellency from micro-post-array depends on the geometric distribution of micro-posts over the surface, the surface free energy of the micro-post structures is important in maintaining the hydrophobic characteristics over the surface^4^. The micro-post arrays can easily be produced on solid surfaces such as silicon wafers. In addition, the micro-post array topology created on silicon wafers can be replicated over soft material surfaces such as polydimethylsiloxane (PDMS), which can be used for self-cleaning applications^5^. The micro-post pillar spacing and maintaining hydrophobicity over the surface become critically important for self-cleaning applications. This is because the low-dimensional dust particles can reside in the spacing of micro-post gaps while resisting mitigation from the surface. One of the methods to increase surface hydrophobicity on micro-post arrays is to introduce a coating layer with low surface energy materials such as functionalized silica nano-particles. Although hydrophobizing of micro-post array enhances liquid droplet mobility on the surface, the droplet mobility can be further enhanced by reducing the interfacial contact area and three-phase contact line over the surface of micro-post arrays. This can be possible via altering the micro-post gap spacing towards increasing the interfacial air-droplet fluid contact length. However, for self-cleaning applications, the possibility of the number of particles (such as dust) residing in the micro-post gaps increases as the micro-post gap spacing increases. This is because the three-phase contact line between the droplet fluid and gap becomes larger, i.e. dust surfaces contribute to the extension of the contact line. This can cause droplet fluid to spread over the dust surface within the gap width. Hence, spreading creates an adverse effect on the droplet mobility and the amount of dust being mitigated from the micro-post array surface becomes less. Consequently, the investigation into the effect of configurations of micro-post array gap spacing on droplet mobility and dust mitigation becomes essential.

Considerable research studies have been reported exploring micro-post arrays and surface water repellency. The processes, in general, involve multi-steps towards creating the surfaces with hydrophobic micro-post arrays, which also depend on the applications. For metallic micro-post structures that are used for high-temperature applications can be produced through surface deposition of multi-layer coatings including Cu/Cr coatings^6^. The wettability of metallic micro-post arrays can be improved through the proper setting of the geometric topology of micro-posts. The dual-scale hierarchical surfaces could be one of the alternative methods for creating the Cassie & Baxter wetting states over the surfaces. Hence, micro-poles having ripple-like nanopatterns give rise to superhydrophobic states^7^. Moreover, a lithographic technique can be used to decorate substrate material surfaces by micro-post arrays. The lithographic technique provides high dimensional accuracy making geometrically desired micro-post arrays. Moreover, dimensional accuracy becomes critical in microbiologic research such as microcontact printing for patterning proteins on substrates^8^. The shape and size of micro-post arrays play an important role in the micro-channel flow towards reducing the pressure drop via lowering frictional drag^9,10^. In addition, micro-post arrays can be impregnated by a fluid while producing lubricant surfaces, which become critically important for medical applications such as artificial hip joints^11^. Micro-post arrays also find various applications in heat transfer enhancement in various applications such as fuel cells. This is because of extended surfaces within the channel wall and capillary effect created around micro-post wicks^12,13^ as well as liquid droplet formation and behavior, such as detachment and removal from fuel-channel wall, are closely related to fuel cell performance^14^. The geometric shape of array posts influences droplet detachment time and mobility inside the fuel-cell channels^15^. The influence of micro-post array geometric configuration, in terms of roughness parameter, remains significant on improved droplet sliding motion over oil-impregnated surfaces^16^. However, self-cleaning applications, particularly droplet mobility enhancement over dusty micro-post array surfaces need further attention for improved dust mitigation rates from the surface.

On the other hand, rolling liquid droplets over the textured hydrophobic surfaces are faced retarding effects due to irregular texture patterns; in which, droplet motion slows down. The droplet dynamics and droplet fluid infusion over solid particles, such as dust particles, play a major role in the self-cleaning of surfaces by water droplets. To accelerate droplet motion over dusty surfaces, rolling of the droplet is required rather than droplet sliding motion. Droplet rolling can be enhanced by creating sufficient air gaps between pillars and droplet fluid at the interface of micro-post arrays. In this case, air gaps act like a cushion at the interface reducing droplet pinning over the surfaces^17^. It is demonstrated that the interfacial air cushion can be created via bubbles inside the droplet fluid, which notably contribute to droplet rolling enhancement^18^. Although droplet rolling over hydrophobic dusty surfaces was presented previously^18,19^, the influence of texture characteristics on the removal of dust from hydrophobized micro-post array surfaces was left for future study. Moreover, achieving cleaning over the entire hydrophobic surface becomes challenging. Hydrophobized micro-post arrays have uniform texture characteristics, which result in uniform texture morphology over the surface. Altering micro-post spacing can provide different texture topology over micro-post arrays surface. This can influence droplet mobility over dusty hydrophobic micro-post surfaces. Hence, in the present study, the dynamics of rolling water droplets over hydrophobized micro-post array surfaces are examined. The study is extended to include dust mitigation from hydrophobized micro-post arrays surfaces having various array spacings. Experiments and numerical simulations are carried out to determine energy losses related to droplet rolling over clean and dusty micro-post array surfaces. Micro-post array surfaces are hydrophobized via deposition of functionalized nano-silica particles. A high-speed recording system and tracker program are utilized to monitor and evaluating droplet motion over the surfaces.

## Numerical

The rolling dynamics of a droplet on a micro-post array surface are considered adopting the Navier–Stokes’ equation. Droplet fluid (water) is considered to be incompressible and Newtonian while the surrounding air is considered to be weakly-compressible due to the low Mach number (i.e. $$Ma<0.3)$$ associated with the airflow created during droplet rolling. Hence, the simulation of droplet rolling is conducted via using the continuity and momentum equations as follows:

1 $$\frac{\partial \rho }{{\partial t}} + \nabla \cdot \left( {\rho \overline{v}} \right) = 0$$

The momentum equation yields:

2 $$\rho \frac{{\partial \overline{v} }}{\partial t} + \rho \left( {\overline{v} \cdot \nabla } \right)\overline{v} = \nabla \cdot \left[ { - p\overline{I} + \mu \left( {\nabla \overline{v} + \left( {\nabla \overline{v} } \right)^{T} } \right) - \left( {\frac{2\mu }{3} - \kappa } \right)\left( {\nabla \cdot \overline{v} } \right)\overline{I} } \right] + \rho \overline{g} + \gamma k_{v} \delta \overline{n} + \overline{F}_{fr}$$

here: $\rho$ is density, $\overline{v }$ is velocity vector, $\overline{g }$ is acceleration vector due to gravity, $p$ is gauge pressure, $\gamma$ is surface tension, $\mu$ is dynamic viscosity, $\kappa$ is the volume viscosity, ${k}_{v}=\frac{\overline{n} }{\left|\overline{n }\right|}$ is curvature where $\overline{n }$ is the unit normal vector at the water–air interface, $\delta$ is Dirac-Delta function, $\overline{F}_{fr}$ is friction source term, and $t$ is time.

The rolling droplet and micro-post array surface are considered to be in stagnant air ambient. To model the rolling droplet model, the conserved level-set two-phase (liquid for water and gas for air) flow model is adopted (Eq. 3), i.e.:

3 $$\frac{\partial \phi }{\partial t}+\overline{v }\cdot \nabla \phi ={\gamma }_{r}\cdot \nabla \cdot \left({\varepsilon }_{ls}\nabla \phi -\phi (1-\phi )\frac{\nabla \phi }{\left|\nabla \phi \right|}\right)$$

here: $\phi$ is a level-set function, $\overline{v }$ is velocity vector, ${\gamma }_{r}$ is re-initialization parameter, ${\varepsilon }_{ls}$ is the parameter that controls interface thickness, and $t$ is time. With the level-set function, properties such as density, viscosity, contact resistance, and thermal conductivity of air and water are scaled according to mixtures rules. Hence, the density can be written as $\rho ={\rho }_{2}+({\rho }_{1}-{\rho }_{2})\cdot {\phi }_{s}$.

### Initial conditions

**Figure 1:**
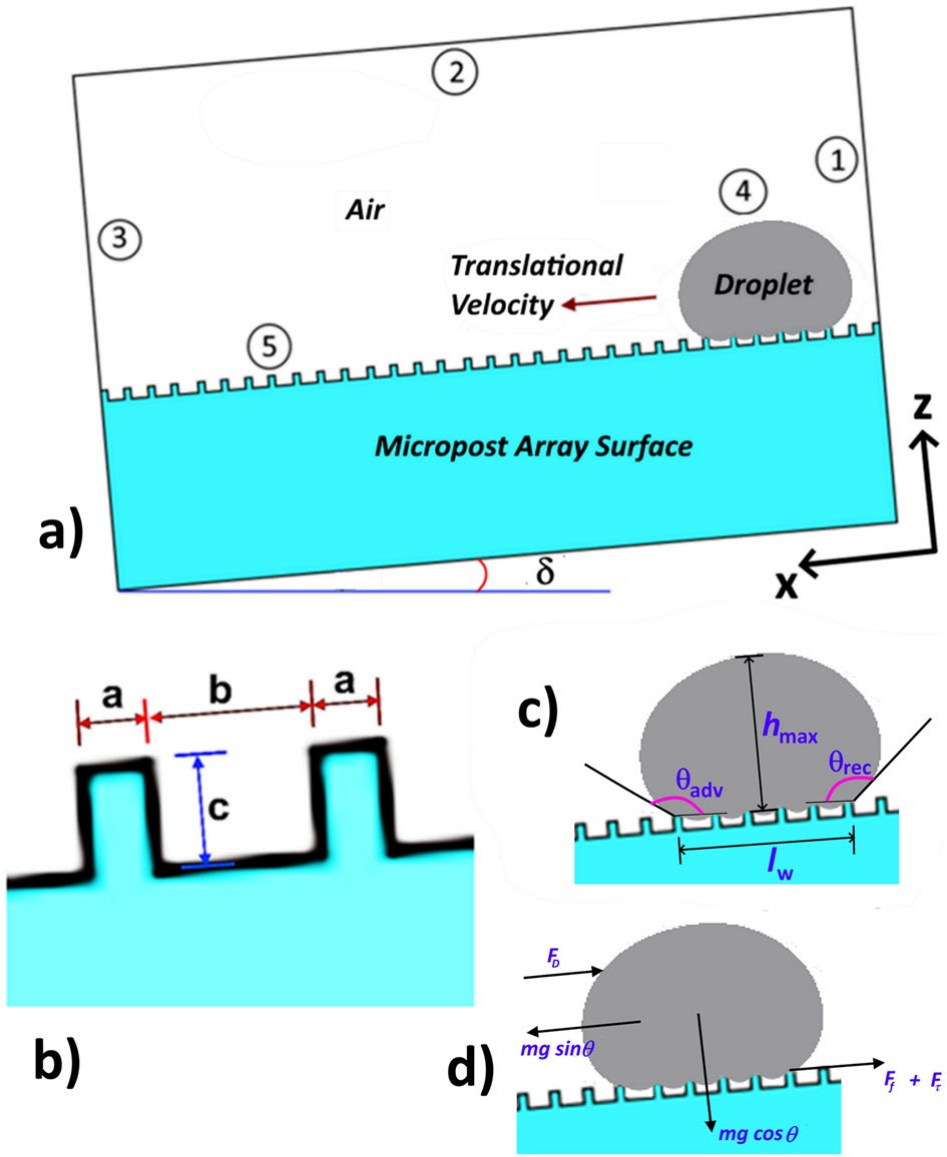
(**a**) Five boundary conditions are adopted for the rolling droplet down the micro-post array surface. (**b**) Geometrical representation of the micro-post array, (**c**) droplet geometrical dimensions, (**d**) forces acting on droplet geometry. The pressure outlet is defined at boundaries 1, 2, and 3. Boundary 4 resembles the interface between the droplet and surrounding, which is free to deform. Boundary 5 is the wetted wall at which the contact angle and adhesive forces are defined.

As depicted in Fig. 1, the droplet is considered to be initially at rest and then rolls down along the inclined micro-post array surface under the gravitational influence. Droplet initial size and location on the surface are defined according to the experimental data. Hence, the initial conditions used for the current analysis are expressed as:

4 $$\overline{v} \left( {x,y,z,0} \right) = \left\{ {\begin{array}{*{20}l} 0 \hfill & {for\;drople} \hfill \\ 0 \hfill & {for\;air} \hfill \\ \end{array} } \right.$$

5 $$p\left( {x,y,z,0} \right) = \left\{ {\begin{array}{*{20}l} {\frac{2\gamma }{R}} \hfill & {for\;droplet} \hfill \\ 0 \hfill & {for\;air} \hfill \\ \end{array} } \right.$$

6 $$\phi_{s} \left( {x,y,z,0} \right) = \left\{ {\begin{array}{*{20}l} 1 \hfill & {{\text{for}}\;{\text{droplet}}} \hfill \\ {0.5} \hfill & {{\text{for}}\;{\text{interface}}} \hfill \\ 0 \hfill & {{\text{for}}\;{\text{air}}} \hfill \\ \end{array} } \right.$$

### Boundary conditions

For fluid flow, the boundaries at locations 1, 2 and 3 (Fig. 1) present the pressure outlet conditions. The interfacial boundary is defined at location 4 (Fig. 1) in between the droplet outer surface and surrounding air, and it is considered to be free to deform within the computational domain. Boundary 5 (Fig. 1) is considered to be a wetted wall in which the dynamic contact angle between droplet, air, and the surface is defined in line with the previous studies^20,21^. In addition, experimentally obtained adhesion/frictional forces are defined at boundary 5 from which droplet motion is inhibited by the resistive interfacial forces. Thus, the boundary conditions used can be expressed as:

7 $$p\left(x,y,z,t\right)=0(pressure\;outlet)$$

8 $$\overline{v} \left( {x,y,z,t} \right) \cdot \overline{n}_{wall} = 0\left( {slip\;at\;wetted\;wall} \right)$$

here, $\overline{n}_{wall}$ is a unit normal vector at the wetted wall.

9 $$F_{fr} = \gamma \left( {\overline{n}_{wall} - \overline{n} \cos \theta_{d} } \right)\delta - \frac{\mu }{\beta }\overline{v} ({\Gamma },t) + F_{ad} \left( {friction\;force\;at\;a\;wetted\;wall} \right)$$

here, $\overline{n }$ is a unit normal vector at the water–air interface, ${\theta }_{d}$ is the dynamic contact angle, $\beta$ is slipped length at the wetted wall and $\Gamma$ denotes a boundary. For numerical calculations, a suitable choice is $\beta =\frac{h}{5}$, where $h$ is the edge length of the mesh element.

A slip boundary is adopted at droplet and air interface; in which case, shear stresses on droplet and air regions are considered to be the same order, i.e., no boundary layer is developed. Hence, the following boundary condition on droplet free surface at the liquid–air interface is introduced:

10 $$\left( { - pI + \mu \left( {\nabla \overline{v} + \left( {\nabla \overline{v} } \right)^{T} } \right)} \right)\overline{n} = 0$$

Moreover, no fluid penetration occurs from the surrounding air; hence:

11 $$\overline{v} \cdot \overline{n} = 0;\;\overline{K} - \left( {\overline{K} \cdot \overline{n} } \right) = 0$$

where, $\overline{K} = \mu \left( {\nabla \overline{v} + \left( {\nabla \overline{v} } \right)^{T} } \right)\overline{n}$. Boundary 5 is the wetted wall at which the contact angle and adhesive forces are defined.

### Numerical implementation

COMSOL multi-physics code^22^ is used to model the dynamics of a rolling droplet on the micro-post array surface incorporating the initial and the boundary conditions presented through Eqs. (1)–(15). The governing equations are presented in the cartesian coordinate system, which enabled to build of the numerical case for 3D simulations. Moreover, the numerical accuracy of the scheme is limited by the time step size, mesh size and level-set parameters adopted. Hence, time steps as small as 10^–8^ s are adopted for the numerical solution ensuring the convergence of time derivatives. The second-order Euler backward difference scheme is used to discretize the time derivatives in the flow and energy equations. With the level-set approach, the droplet interface is treated implicitly such that numerical convergence is achieved with a regular mesh of sufficient density (shown as Fig. S1 in the “Supplement”). However, the level-set parameters (${\gamma }_{r}$ and ${\varepsilon }_{ls}$) need to be properly set so that the movement of the droplet can be effectively captured within the fixed mesh. Based on the initial tests and the practice, the re-initialization parameter (${\gamma }_{r}$) is set to be equal to the maximum velocity of flow (0.6 m/s); while the interface thickness (${\varepsilon }_{ls}$) is taken as half of the maximum element (edge) length. As demonstrated in Fig. S2 (in the “Supplement”), the grid-independent solution for the 40 µL droplet and gap spacing of b = 50 µm (Fig. 1b) after a total duration of 21 ms is obtained with 1,279,003 tetrahedral elements having an average quality of 0.94. Furthermore, mass is conserved during the analysis by ensuring that the change in mass during the impact of the droplet is negligible.

## Experimental

Silicon wafers of 1 mm thickness were used to create micro-post arrays texture over the surfaces by using the lithographic method. Micro-post arrays of 10 μm, 25 μm, and 50 μm gap spacing were produced on silicon wafer surfaces. Since the silicon wafers were opaque to the UV visible spectrum, micro-post array texture topology was replicated by polydimethylsiloxane (PDMS). In order to hydrophobizing micro-post arrays surfaces, functionalized silicon nanoparticles were deposited via dip-coating technique. The silica nanoparticles were prepared in line with the early study^23^. The silica nanoparticles were synthesized incorporating tetraethyl orthosilicate (TEOS), isobuthytrimethoxysilane (OTES), ethanol, and ammonium hydroxide. In this case, 14.4 mL of ethanol, 1 mL of de-ionized water of 18.2 MΩ resistivities, and 25 mL of ammonium hydroxide were stirred for 20 min. The resulting solution was mixed with 1.5 mL TEOS diluted in 4 mL of ethanol and stirring was continued for 45 min. Later, a modifier silane was added in a molar ratio of 3:4 and the final mixture was stirred for 10 h. The dip-coating method was adopted for coating functionalized silica nanoparticles onto the surface of the micro-post array. The micro-post arrays were immersed in the final solution using the dip-coating unit (Biolin Scientific). The wetting state of the hydrophobized PDMS surfaces is assessed incorporating the contact angle measurements. A goniometer (Kyowa, model—DM 501) was used and high precision drop shape analysis was used for evaluating the droplet contact angles^24^. The hydrophobized micro-post array surface demonstrated a uniform wetting state over the surface and the contact angle measured over the hydrophobized surface was found to be almost independent of micro-post array gap spacing. Hence, the contact angle of 150° ± 3° with the hysteresis of 3° ± 1° was measured. The roll-off angle of the droplet was measured using 7 μL droplet and is found to be 3.8° ± 0.2°. The low hysteresis of the contact angle was associated with the nano-size silica particles, which created like Lotus effect on the surface. Moreover, low contact angle hysteresis was also reported in the early study^25^. A JEOL 6460 SEM (scanning electron microscope) with a magnification of 10× to 300,000× was used for imaging the micro-post array topology before and after the coating. A high-speed video-recording facility (Speed Sense 9040) was used to record the rolling droplet motion over the surfaces. The video recording was realized at 5000 frames-per-second (fps) with the pixel size of 14 µm × 14 µm and having the resolution of 1280 × 800 pixels. A tracker program^26^ was used to quantify the recorded data in terms of droplet rolling and transitional velocities. Video recording experiments were repeated ten times and the error estimated was about 2.7%. It is worth to mention that the rolling droplet shape slightly differs for each repeat and this creates a considerably small influence on the droplet rolling velocity. The uncertainty (*σ*_*u*_) involved with the measurements were evaluated through^27^:

12 $$\sigma _{u} = \sqrt {\int\limits_{{x_{o} }}^{{x_{m} }} {\left( {x - x_{e} } \right)^{2} \chi \left( x \right)dx} }$$

here, *x*_*e*_ corresponds to the mean value of the data (*x*), *m* is the number of points in the data-set, and $\chi$(*x*) being the probability distribution function. The function $\chi$(*x*) was determined from an instant correlation plane. Later, it was fitted into a Gaussian function to evaluate the corresponding Gaussian diameter. The uncertainty was evaluated by adopting a least-squared-Gaussian-fit technique and later it was normalized with the pixel numbers contributing to the cross-correlation peak. In addition, the bias error was about 0.02 pixels, which was based on the complexity involved in quantifying extremely small peaks in the Gaussian function. The uncertainty was estimated at 2.9%.

Dust was gathered from PV panel surfaces via using soft brushers in the close region of Dammam in Saudi Arabia and dust was stored in tightly sealed containers. Dust particles were deposited forming a layer over hydrophobized micro-post arrays using metallic meshes towards achieving the uniform dust layer thickness. The dust layer thickness is kept at about 300 μm, which was the same order of averaged dust layer thickness on PV panel surfaces over six months period in the Dammam area in Saudi Arabia.

## Results and discussion

Droplet mobility assessment over hydrophobized micro-post array surfaces is considered and dust mitigation by rolling droplet is evaluated. Droplet motion is monitored and characterized by using a high-speed camera and a tracker program in terms of rolling, sliding, and translational velocities of the droplet, droplet wetting over the surface, and maximum droplet height. Droplet behavior is simulated numerically adopting the experimental conditions.

### PDMS replication of micro-post arrays and surface hydrophobizing

**Figure 2:**
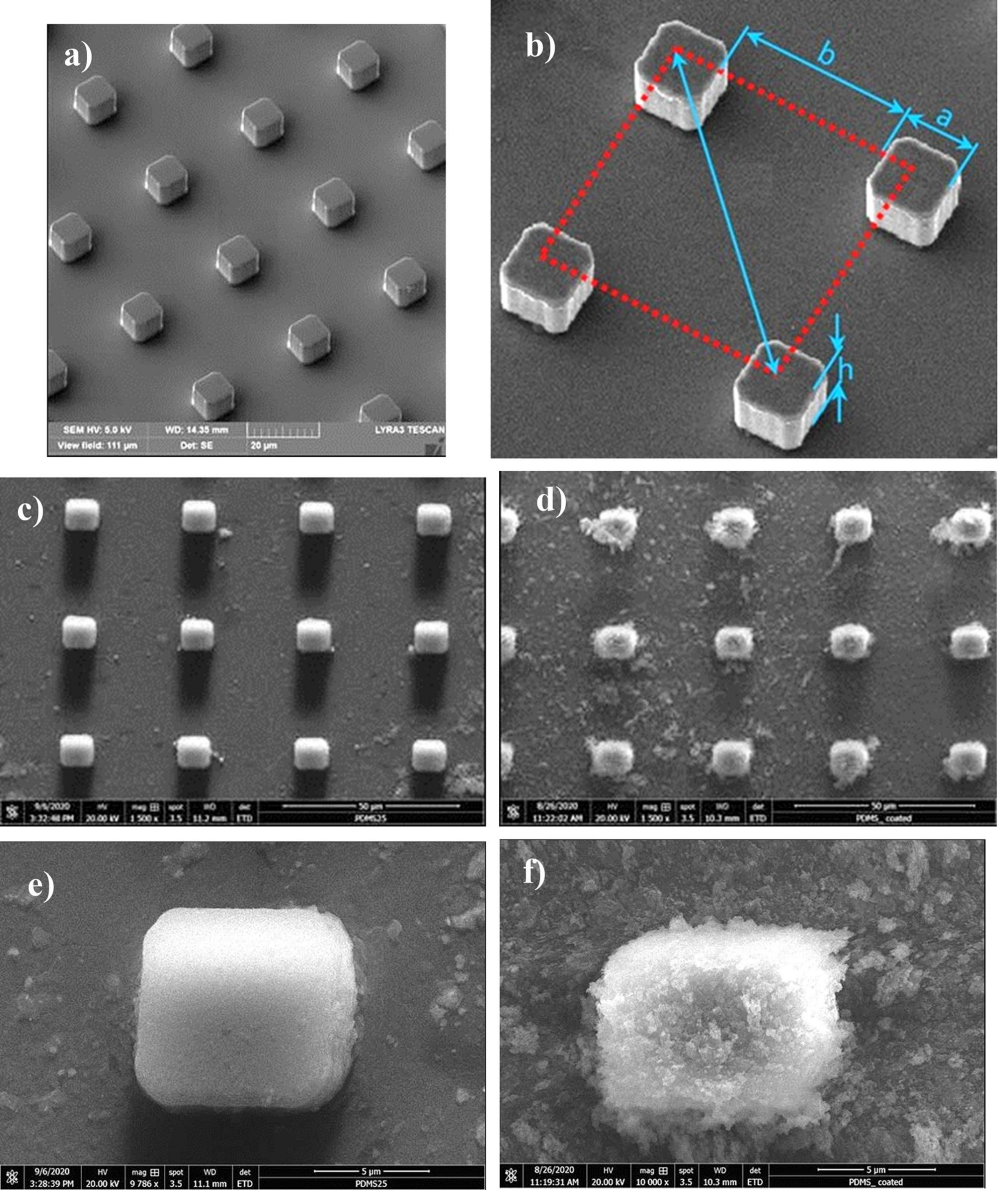
SEM micrographs of micro-post array surface: (**a**) micro-post on a silicon wafer, (**b**) micro-post sizing, (**c**) replicated micro-post on PDMS, (**d**) functionalized silica nanoparticles deposited replicated surface, (**e**) replicated single micro-post, and (**f**) functionalized silica nanoparticles deposited single micro-post and surroundings.

Micro-post arrays are created over silicon wafers and later replicated over polydimethylsiloxane (PDMS) wafer surfaces. Figure 2a–c shows micrographs of micro-post arrays formed on a silicon wafer and replicated micro-post arrays on PDMS wafer while Fig. 2d–f show a close view of PDMS micro-posts before and after coating. The replicated micro-post arrays on PDMS are identical to micro-post arrays formed on a silicon wafer. In addition, the corners of the micro-post is not rounded after replication (Fig. 2e). The area ratio $\left( {\phi_{s} = \frac{{A_{solid} }}{{A_{\Pr ojected} }}} \right)$ determining the fraction of post area over the projected area for the micro-post arrays is (Fig. 2b):

13 $$\phi_{s} = \frac{{a^{2} }}{{\left( {a + b} \right)^{2} }}$$

here, *a* is the length of the square micro-post and *b* is the gap length (spacing between two-consecutive micro-posts). In addition, the area ratio of the total micro-posts surface over the projected area yields:

14 $$r = \frac{{\left( {a + b} \right)^{2} + 4ah}}{{\left( {a + b} \right)^{2} }} = 1 + \frac{4ah}{{\left( {a + b} \right)^{2} }}$$

here, *h* is the height of the micro-post. The droplet fluid is expected not to spread over the micro-post array surface during rolling. The wettability of the plain (not textured) PDMS surface is assessed by estimating the spreading factor of the droplet fluid over the surface. The spreading factor for plain PDMS surface is: ${S}_{s-w}={\gamma }_{w}-{\gamma }_{s}-{\gamma }_{s-w}$, here γ_w_ is droplet fluid surface tension (water), and γ_s_ is the surface free energy of Polydimethylsiloxane, and ${\gamma }_{s-w}$ is the interfacial tension between droplet liquid and Polydimethylsiloxane. The surface free energy of Polydimethylsiloxane is 21.3 mJ/m^2^^28^ and the interfacial tension between droplet fluid and Polydimethylsiloxane is 40 mJ/m^2^^29^. This gives rise to ${S}_{s-w}$ in the order of ${S}_{sw(a)}=10.7$ mJ/m^2^, which demonstrates that the droplet fluid can spread over the plain Polydimethylsiloxane surface (${S}_{sw(a)}> 0$). However, creating the micro-post array on the PDMS surface, via the replication method, can alter the spreading rate of the droplet fluid over the surface because of reduced interfacial resistance under the influence of the roughness parameter. In addition, uncross-linked chains can affect the droplet dynamics on the PDMS surface^30^; however, this effect is assumed to be negligible in the present study. The condition for partial-spreading of droplet fluid over micro-post surface satisfies: ${S}_{s-w}<-{\gamma }_{w}\frac{(r-1)}{(r-{\phi }_{s})}$^31^. Since three different spacing of micro-post arrays (*b* = 10 μm, 25 μm, and 50 μm) are replicated while keeping other dimensions of micro-post arrays (*a* = 10 μm and *h* = 10 μm) are same, the droplet fluid spreading factor over the different space setting of micro-post array surface changes. Inserting the values for *r* and *ϕ*_*s*_ from Eqs. (13) and (14), the spreading factor (${S}_{s-w}$) takes the values − 0.04114 for *b* = 10 μm, − 0.01889 for *b* = 25 μm, and − 0.00738 for *b* = 50 μm. Hence, droplet liquid can partially spread over the micro-post array surface. To eliminate the partial spreading of the droplet liquid over the surface, micro-post array surfaces are hydrophobized through the deposition of functionalized silica nanoparticles. Figure 2d,f show functionalized silica nanoparticles deposited micro-post array surface as formed on PDMS surface. Silica nanoparticles uniformly cover the surfaces of micro-posts and gap spacings over micro-post arrays. The nanoparticles are spherical and almost 30 nm in size. They agglomerate over the deposited surface of micro-posts and gap spacing forming fine size peaks and valleys. This is because of the condensing monomers, which suppress the nucleation rate and lowers the number of new nuclei formation during the synthesizing cycle of the nano-silica particles^32,33^. As the condensation takes place at high rates, it results in adhesion and agglomeration of the silica particles over the surface. The average roughness of nanoparticles deposited surface is about 160 nm. The water droplet contact angle is measured is about 150° ± 3° and the hysteresis is about 3° ± 1°. It should be noted that high-precision drop shape analysis is adopted to measure the droplet contact angle^24^. Hence, coating of micro-post arrays surface results in a hydrophobic wetting state.

### Droplet rolling over hydrophobic micro-post arrays

The rotational kinetic energy of the rolling droplet over inclined hydrophobic surfaces is influenced by the forces of pinning (under surface tension), air drag, and interfacial shear (created over the wetted surface)^34^. The pinning force is proportional to the droplet wetting diameter (*D*_*w*_), the solid fraction (*ϕ*_*s*_), droplet fluid surface tension (*γ*), and advancing (*θ*_*A*_) and receding (*θ*_*R*_) angles during rolling. It takes the form: ${F}_{ad}=\frac{24}{{\pi }^{3}}\gamma {\phi }_{s}D(cos{\theta }_{R}-cos{\theta }_{A})$^34^. The air-drag force acting over rolling droplets can be formulated via ${D}_{a}\cong 1/2{C}_{d}{\rho }_{a}{A}_{c}{U}_{f}^{2}$, here, *C*_*d*_ is the drag coefficient and air velocity is *U*_*f*_, which can be considered as rolling droplet translational velocity (*V*_*d*_)^35^. The interfacial shear force created over the wedded area can be simplified to take the form of: ${F}_{\tau }={A}_{w}(\mu \frac{d{V}_{d}}{dy})$, here *A*_*w*_ is the wetted area, *μ* is fluid viscosity, *y* is distance normal to the wetted area. Droplet wobbling during rolling gives rise to volume deformation of the droplet during its transition over the surface. This causes droplet kinetic energy dissipation over the rolling surface. Moreover, the force balance yields the rotational velocity (*ω*) of the droplet on the inclined hydrophobic surface^34^:

15 $$\omega = \sqrt {\left( {\frac{{\frac{5}{2mR}\left( {mg\sin \delta - \frac{24}{{\pi^{3} }}\gamma \phi_{s} \left( {\cos \theta_{R} - \cos \theta_{A} } \right) - \mu A_{w} \frac{\partial u}{{\partial y}} - \mu_{f} mg} \right)}}{{1 + \frac{5}{4m}C_{d} \rho_{a} A_{c} R}}} \right)}$$

where *m* is droplet mass, *R* is droplet radius, *δ* is the inclination angle of the hydrophobic surface and *μ*_*f*_ is friction coefficient between the droplet and the surface. Hence, droplet size, advancing and receding angles of the droplet, interfacial resistance, and drag coefficient influence the droplet rotational velocity. In addition, the rolling droplet suffers from wobbling because of gravity, which in turn causes extra work done due to the geometric deformation of the rolling droplet. It is worth mentioning that for small droplets (comparable to capillary length (${{\kappa }_{c}}^{-1}$), ${\kappa }_{c}^{-1}=\sqrt{\frac{\gamma }{\rho g}}$), the puddling ceases and droplet rolls like a spherical solid body^36,37^. The volume deformation alters advancing and receding angles of the rolling droplet. Hence, the pinning force can be influenced indirectly by the droplet wobbling. This situation becomes critical as the rolling droplet size increases. In the case of micro-post arrays, air trapped in the spacing between the micro-posts acts like an air cushion while minimizing the droplet fluid contacting at the PDMS surface. Hence, the pinning force and the fluid shear stress over the contacted area are affected by the size of micro-post spacing because of reduced: (1) length of the three-phase-contact line, and (2) interfacial contact area between the micro-post surface and droplet fluid, i.e. air trapped in micro-posts spacing lowers both the contact length and area between droplet fluid and surface. The solid fraction $\left( {\phi_{s} = \frac{{a^{2} }}{{\left( {a + b} \right)^{2} }}} \right)$ reduces as the spacing (*b*) between the micro-posts increases, which results in reduced pinning force. In addition, the projected solid area (*a* + *b*)^2^ reduces by *b*^2^, i.e. $b\ge a$ and the condition $b=a$ gives rise to almost 1/4 reduction of the solid region in the projected area.

Moreover, the wobbling of droplets causes the change of the gravitational center of the droplet with an amount $\lambda$. The difference in the potential energy between puddled and spherical droplets having the same volume is about $\gamma \lambda$ ^*2*^ ≅ *ρgR*^*3*^^38^, here *R* being the droplet radius^38^. The length of droplet liquid contact (*l*_*w*_) over the solid surface for the wobbling droplet is ${l}_{w}\cong \sqrt{R\lambda }$. Hence, the relation $\rho g\lambda \sim \gamma {l}_{w}^{2}/{R}^{3}$ can be written for potential energy difference between spherical and puddled droplets. This gives rise to the contact length of about ${l}_{w}\cong {R}^{2}/\sqrt{\frac{\gamma }{\rho g}}$, which corresponds to the ratio of the square of droplet radius over the capillary length, which is also reported earlier^38^. However, the contact length (${l}_{w}$) reduces by the repeats of the number of micro-posts spacings along the contact length. Hence, the contact length increases because droplet puddling over the surface of the micro-post array becomes less than that corresponding to hydrophobized plane surface. In addition, the force ratio of gravity over the surface tension varies during rotation because of droplet wobbling. This effect can be associated with the rotational Bond number, which is $\frac{\rho {\omega }^{2}{R}^{3}}{8\gamma }$, where *ω* being the droplet rotational velocity. At high rotational velocities, the rotational Bond number increases significantly because of the square relation with the rotational velocity. The droplet wobbling ceases at high rotational Bond numbers^39^. Hence, increasing the droplet rotational velocity lowers droplet wobbling and the difference between the centroidal and the gravitational center of the droplet becomes significantly small. In addition, as the droplet rolls over the hydrophobic surface, the droplet undergoes sliding because of the slip length on the hydrophobic surface. This influences the dynamic pressure generated inside the droplet; hence, the difference between droplet fluid and its ambient pressures varies with time. Nevertheless, this influence becomes negligibly small on the hydrophobic surface^40^.

**Figure 3:**
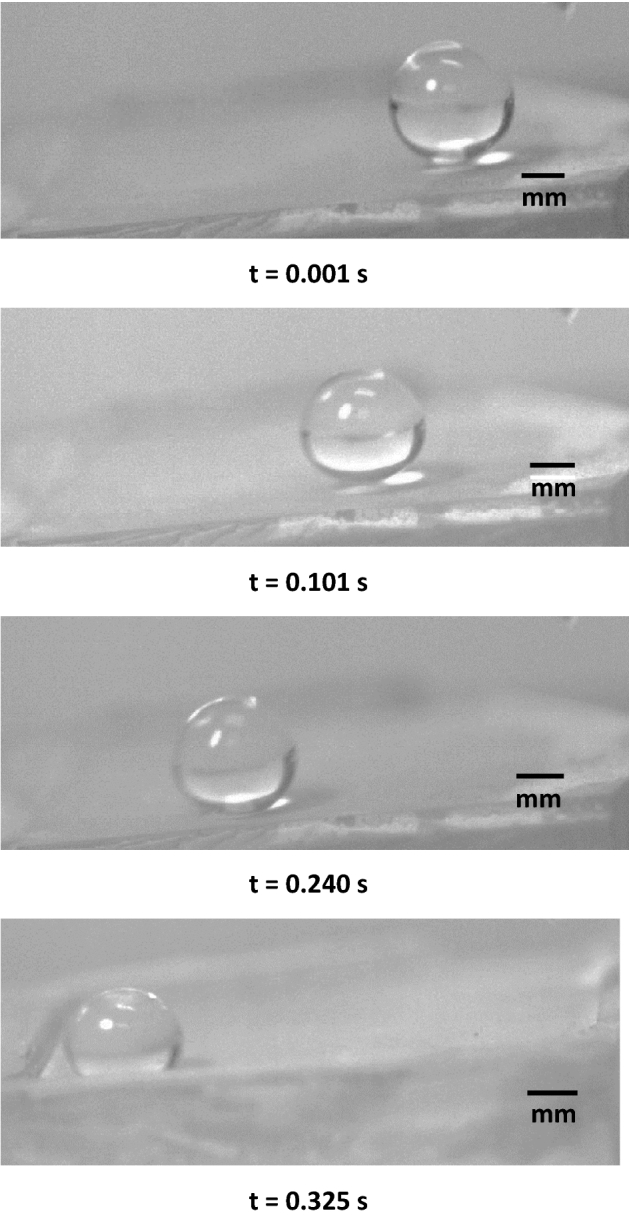
High-speed camera images of a water droplet on micro-post array surface at different durations. Droplet size is 40 μL, inclination angle δ = 5°, and micro-post spacing is 25 μm.

**Figure 4:**
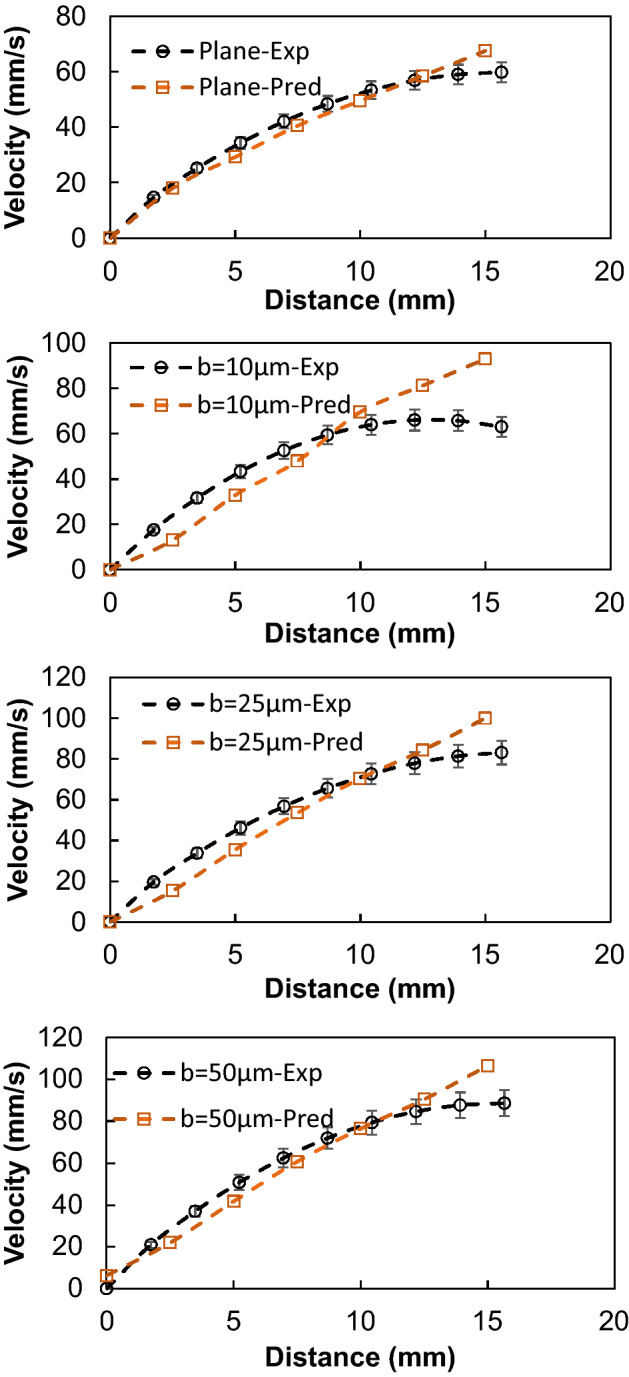
Comparison of droplet velocity obtained from experiments and analytical formulation for various gap spacings. Droplet size is 40 μL, inclination angle δ = 5°.

Figure 3 shows high-speed camera images of the droplet over the inclined micro-post array surface. The droplet wobbling results in a transient change of droplet maximum height and maximum width, which affect the wetting length and advancing and receding angles of the droplet. It is worth to mention that the droplet is formed on the micro-post arrays surface and it is released from the rest by using the micropipette. Upon release, the recording of droplet motion is initiated via high speed-camera. Figure 4 shows droplet translational velocity obtained from experiments and predicted from Eq. (15) after multiplying by rolling droplet hydraulic radius and the ratio of *V*_*d*_/*ωR,* along the inclined surface. It can be observed that analytical solution results in similar droplet velocity to those obtained from the experiment. The differences in droplet velocities are small and can be related to the experimental errors, which is about 5%, and the assumptions made in the analysis, such as constant air-drag force, and uniform surface texture. Moreover, the droplet translational velocity increases almost parabolically with the distance even though the magnitude of velocity changes with micro-post gap spacing. The droplet velocity remains lowest on the plain hydrophobized PDMS surface as compared to the micro-post array surface. The gap spacing influences the droplet translational velocity over the inclined hydrophobized micro-post surface. Increasing gap spacing enhances the droplet velocity, which is more pronounced for the gap space of *b* = 50 μm. However, in the early stage of droplet rolling, the rate of increase of velocity is higher for the large gap spacing. This demonstrates that air trapped within the gap reduces the droplet contact area on the hydrophobized surface, which modifies both the shear effect created over the wetted surface and the lateral component of the surface tension force because of the reduced three-phase contact line.

**Figure 5:**
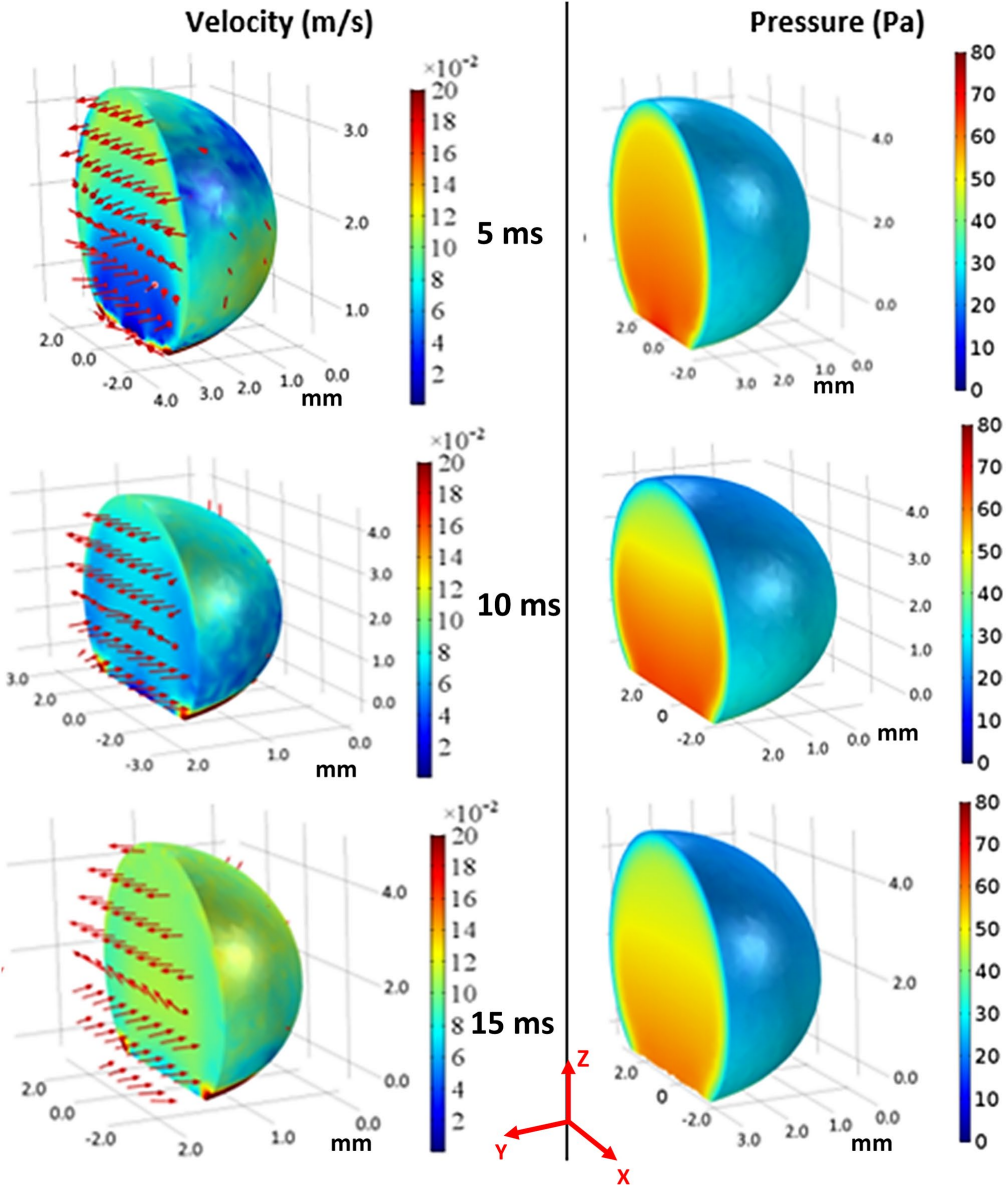
3D velocity field and pressure distributions inside rolling droplet at different durations. Droplet volume is 40 µL, the inclination angle is δ = 5° and micro-post spacing is ${\text{b}}=50\;{\upmu}{\text{m}}$.

**Figure 6:**
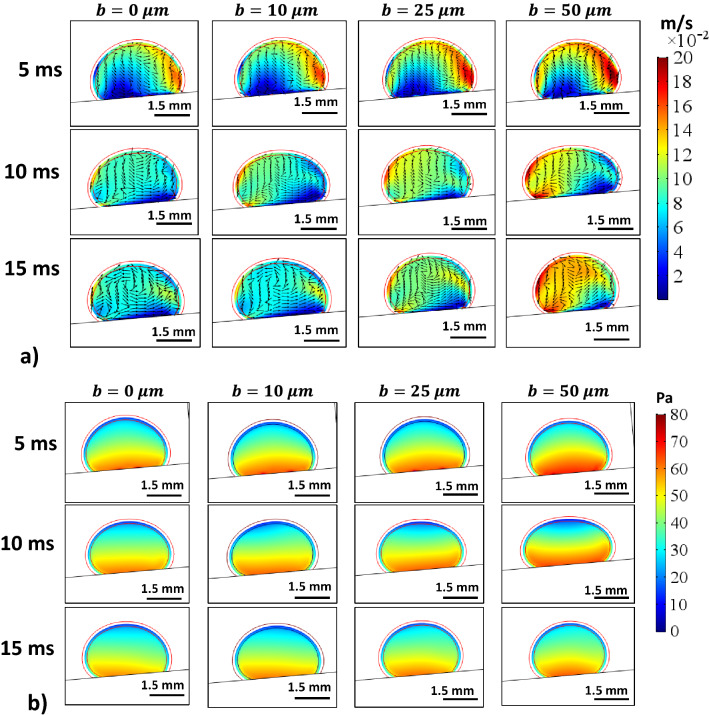
(**a**) Velocity and (**b**) pressure contours in a rolling droplet at different durations for different micro-post spacings. Droplet volume is 40 µL, the inclination angle is δ = 5°. Section cut taken at the cross-sectional plane ($y=0$).

**Figure 7:**
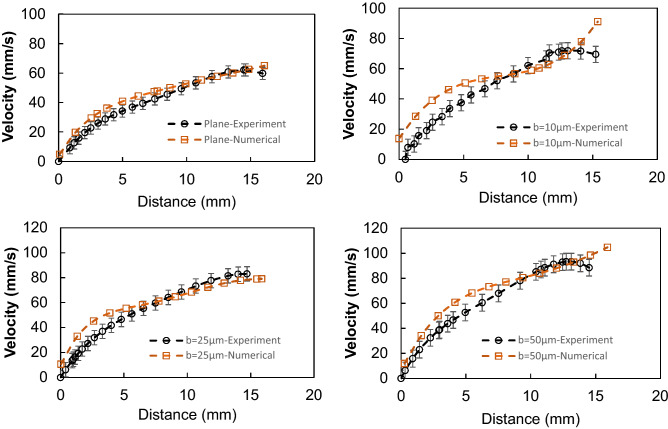
Droplet translational velocity obtained from experiment and simulations for various micro-post gap scrapings. for 40 µL droplet, δ = 5°. Droplet volume is 40 µL, the inclination angle is δ = 5°.

**Figure 8:**
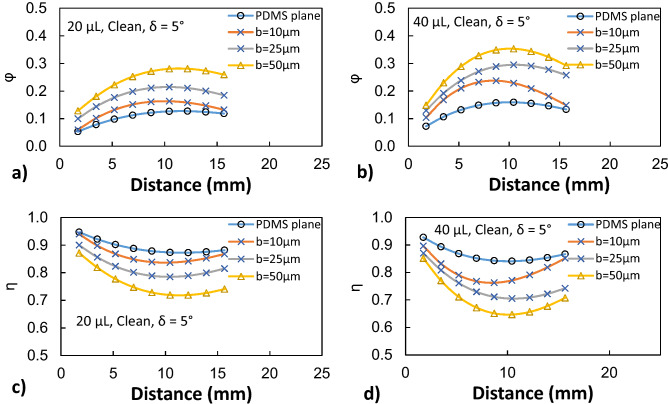
(**a**) Droplet kinetic energy change ratio ($\varphi$) and (**b**) droplet kinetic energy dissipation ratio ($\eta$) for various micro-post gap spacings.

In order to evaluate the velocity inside the droplet fluid when rolling over the hydrophobic PDMS micro-post arrays surfaces, the numerical simulations are carried out adopting the conditions used in the experiments. Figure 5 shows 3-D velocity and pressure contours inside the rolling droplet over the micro-post arrays surface at different times. The maximum flow velocity in the droplet fluid is about 20 mm/s. As the velocity reduces in the droplet fluid, the color changes towards blue in Fig. 5. In the early rolling period (5 ms), in the droplet bottom region (close to droplet fluid and micro-post array interface) velocity reduces. However, with rolling progress (20 ms), velocity within the droplet fluid also increases in this region. Nevertheless, the complex velocity field is created inside droplet fluid and wobbling contributes to flow complexity in the droplet fluid. It is worth mentioning that wobbling causes variation of the maximum droplet height and width. Figure 6 shows (Fig. 6a) velocity and (Fig. 6b) pressure contours inside the cross-section of the droplet for different micro-post arrays gap spacing and plain hydrophobized PDMS surface at various rolling durations, respectively. The circulating flow structures are created in the droplet fluid and the center of circulations changes during the rolling, which causes the development of a complex flow field inside the droplet fluid. However, in the early rolling stages (5 ms), the flow structure formed in the droplet fluid becomes similar for all micro-post spacings considered. As the rolling period progresses, the flow structure changes, and the velocity magnitude close to the interface region attains larger values as compared to the plain PDMS surface as the gap spacing of the micro-post arrays. In addition, the maximum velocity magnitude increases for large gap spacing (b = 50 μm), and the progression of rolling results in the extension of the high-velocity region into the droplet fluid. In this case, increasing gap spacing of micro-post arrays can create air cushions for the droplet fluid at the interfacial while reducing interfacial contact and low resistance for rolling. Figure 7 shows droplet velocity predicted from the numerical simulations and obtained from the experiment via high-speed recorded data, for different micro-post gap spacings. The droplet velocity predicted agrees well with the experimental data. In this case, increasing micro-post gap spacing results in increased droplet velocity. Hence, the trapped air in the micro-post gaps reduces both the droplet fluid contact area and the wetted length over micro-post array surfaces. In order to assess the kinetic energy loss for the rolling droplet over micro-post array surface, the ratio of a kinetic energy change over the potential energy change of the droplet ($\varphi =\frac{{V}_{d}^{2}}{2glsin\delta }$, here *V*_*d*_ is instant droplet velocity, *l* is the instant distance covered by the droplet over the inclined micro-post array surface, and *δ* is the inclination angle of the surface) is evaluated over the inclined micro-post array surface for three droplet volumes. It is worth to mention that the translational velocity composes of the rotational and sliding velocity of the droplet. Since sliding velocity is negligibly small, the translational velocity is incorporated when calculating the droplet kinetic loss. Figure 8a,b show the kinetic energy change ratio ($\varphi$) of the droplet with distance along the inclined micro-post array surface for various micro-post gap spacings and two droplet volumes. The droplet velocity over hydrophobized plain PDMS surface is also provided for comparison. The kinetic energy ratio increases with distance along the inclined micro-post and plain PDMS surfaces. Hence, the kinetic energy ratio increases as the velocity of the droplet increases (Fig. 8a,b). This demonstrates that the loss of droplet kinetic energy, due to the effect of retarding forces (pinning, shear, and air drag), becomes less as the droplet velocity increases over the surface. This is related to droplet wobbling during rolling, which alters advancing and receding angles of the droplet and the wetting length at the interface; in which case the oscillation in the maximum droplet height reduces. The gradient of kinetic energy ratio remains larger immediately after initiation of droplet rolling and it becomes gradual as the distance covered by the rolling droplet increases. Therefore, the rate of kinetic energy dissipation becomes less in the early rolling period over the surface. Increasing gap spacing between the micro-posts results in an increased kinetic energy ratio. This is related to reduced wetting diameter and interfacial area of droplet fluid over micro-post arrays surface, i.e. increasing gap size reduces droplet fluid wetting length and area over the micro-post array surface. Moreover, increasing droplet volume gives rise to an increased kinetic energy ratio for all gap spacings. This indicates that the gravitational inertia causes droplet velocity enhancement for large droplet volumes, which also gives rise to less droplet kinetic energy loss during rolling over the micro-post array surface. Moreover, Fig. 8c,d show droplet kinetic energy dissipation ratio ($\eta =1-\frac{{V}_{d}^{2}}{{V}_{ideal}^{2}}$, where *V*_*ideal*_ is the velocity of a solid droplet having the same volume and without subjecting of the retarding forces, i.e. rolling like a marble) with rolling distance along micro-post array surface for different gap spacing and droplet volumes. The kinetic energy dissipation ratio demonstrates similar behavior to the kinetic energy ratio. In this case, increasing distance along the micro-post surface enhances the kinetic dissipation during rolling. Similarly, increasing micro-post gap spacing reduces the kinetic energy dissipation of the droplet; hence, the maximum kinetic energy dissipation occurs for plain hydrophobic PDMS surface. Increasing droplet volume lowers the kinetic energy loss even though the wetting length and contact area at the interface increase with increasing droplet volume. This is because gravitational inertial force remains significantly larger than the retardation forces acting over the rolling droplet.

### Droplet dynamics and dust on surface

**Figure 9:**
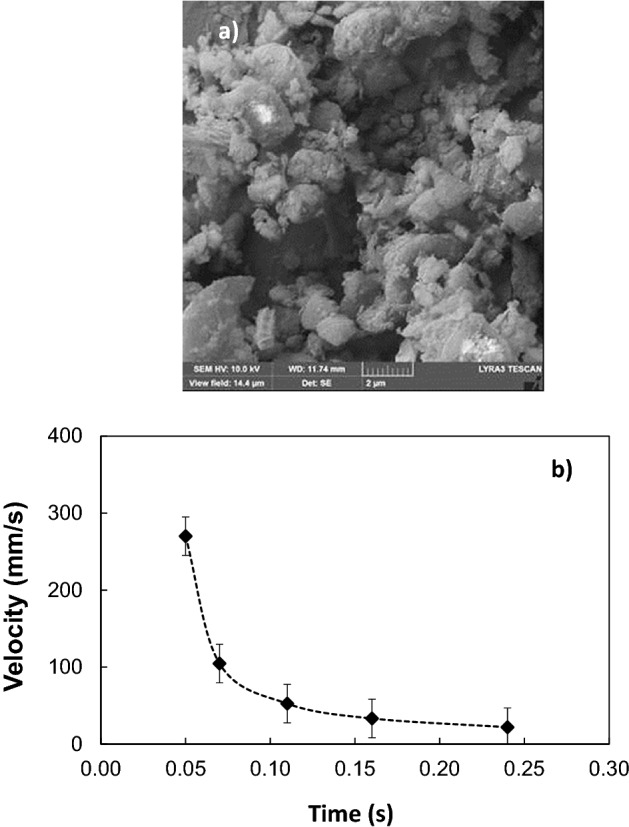
SEM micrographs of dust and infusion velocity of water over dust particle: (**a**) dust particles with various sizes and shapes, and (**b**) infusion velocity of water over dust particle.

**Figure 10:**
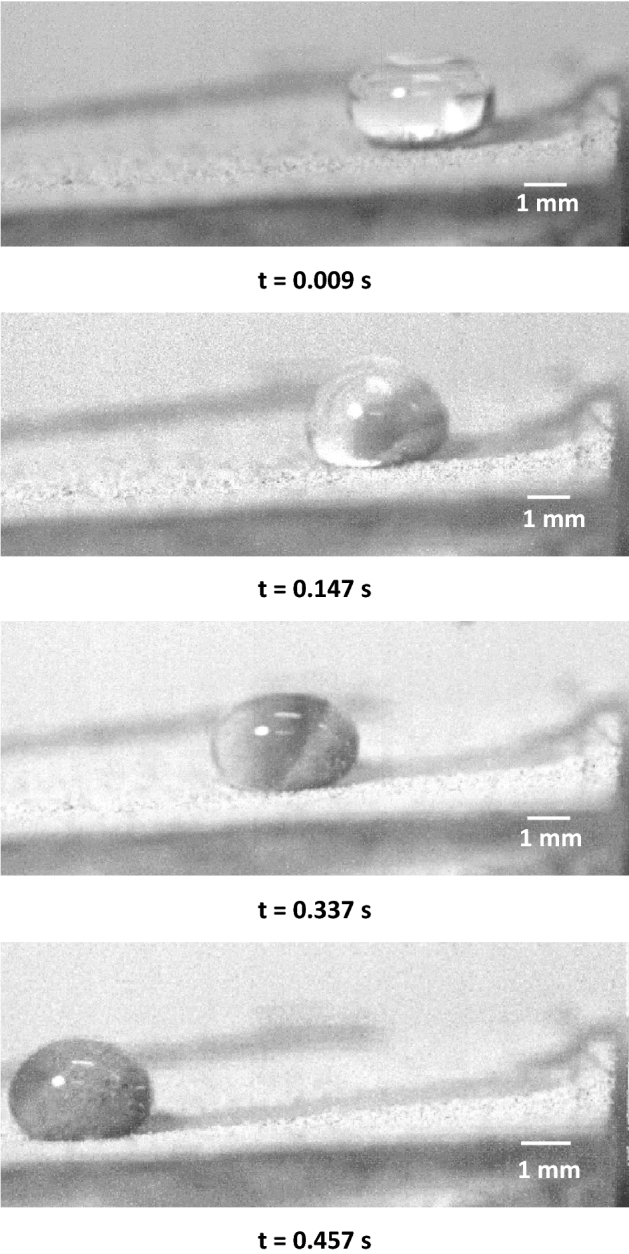
High-speed camera images of water droplets over dusty micro-post array surfaces at different time durations.

**Figure 11:**
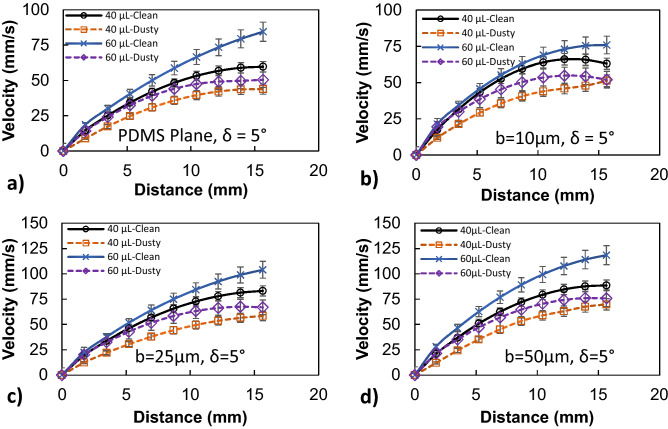
Droplet translational velocity obtained from experiment due to dusty and clean surfaces for two droplet volumes and various micro-post gap spacings.

**Figure 12:**
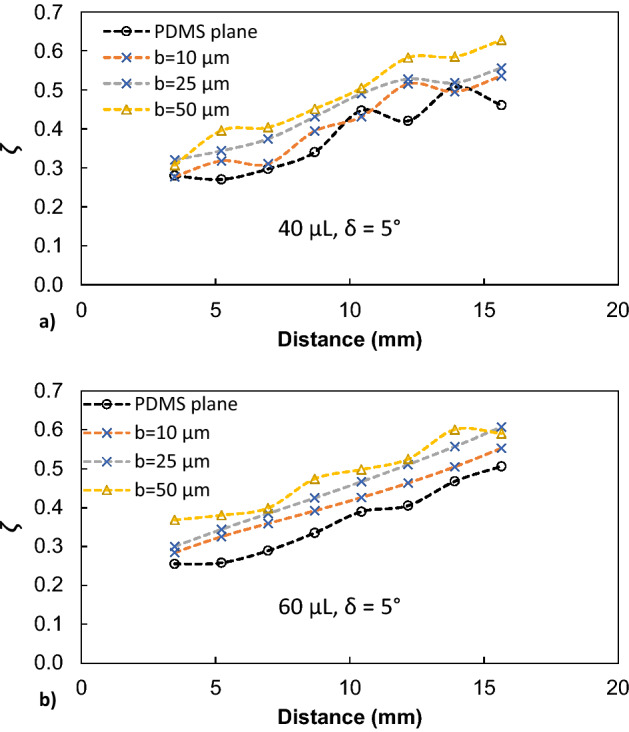
Droplet kinetic energy loss ratio ($\zeta$) for various micro-post gap spacings.

**Figure 13:**
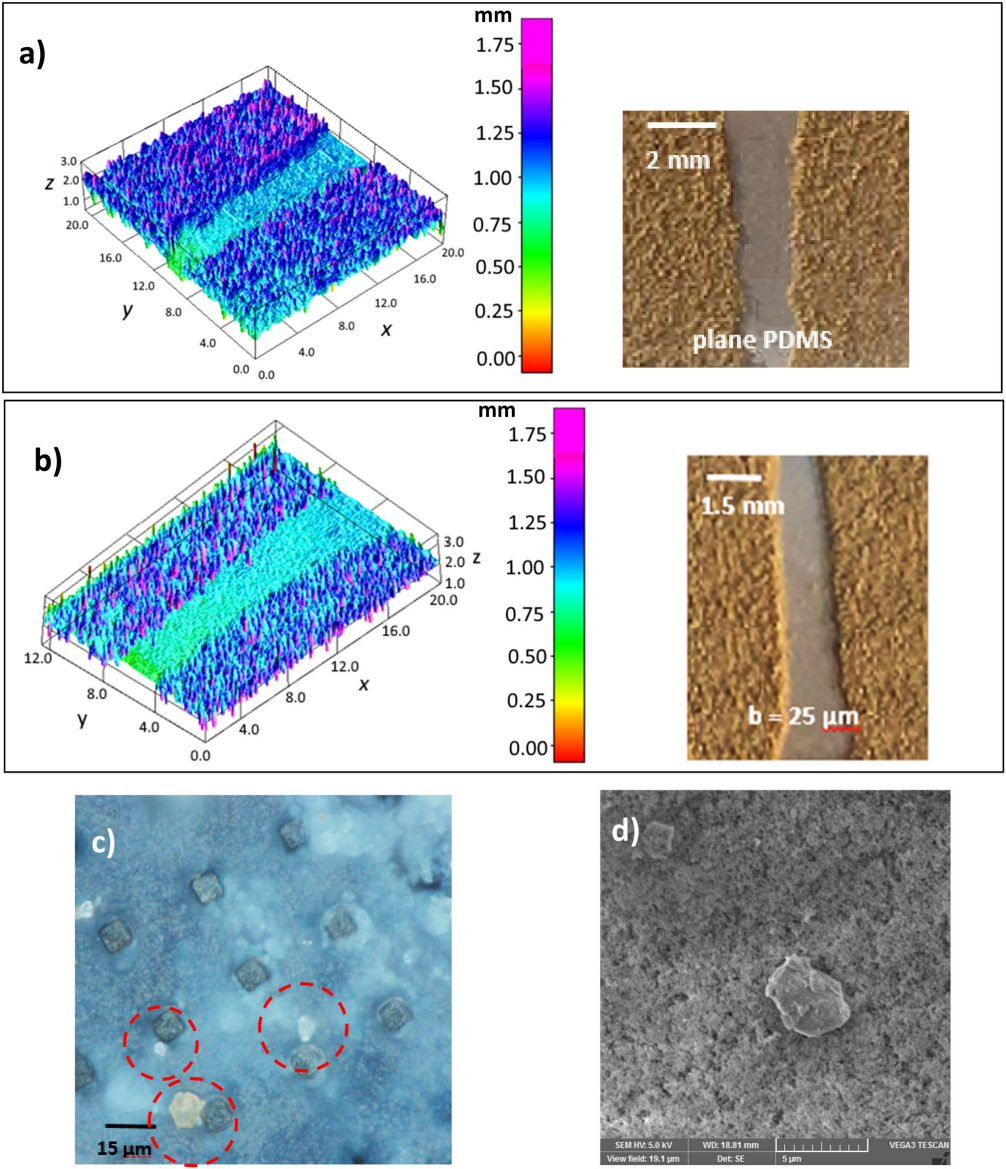
Optical and SEM images of droplet path and dust residues: (**a**) optical image of droplet path on plain PDMS, (**b**) optical image of droplet path on micro-post arrays with 25 μm gap spacing, (**c**) optical image of micro-post array surface within droplet optical path (red dotted circles show dust residues), and (**d**) SEM micrograph of dust residues within droplet path.

Dust is collected from PV panel surfaces via soft brushes and they are uniformly deposited over micro-post array surfaces having a thickness of about 300 μm. The droplet rolling tests are carried out and the droplet motion is recorded accordingly. Dust consists of various sizes of particles, which are composed of various elements. SEM micrograph of dust is shown in Fig. 9a. The average size of dust is about 1.2 μm and its composition consists of mainly Si, Ca, Na, K, Mg, O, Cl (Table 1). Dust composition shows that dust has salt compounds, which can dissolve in water^41^. The droplet fluid (water) infusion over dust remains critical for dust particles to be picked up by rolling droplet fluid^42^. In this case, the coefficient of droplet liquid spreading over the dust surface needs to remain greater than zero for droplet fluid infusion. The coefficient of liquid spreading (${S}_{s-l}$) over dust surface is governed by ${S}_{s-l}={\gamma }_{s}-{\gamma }_{s-l}-{\gamma }_{l}$, here ${\gamma }_{s}$ is the surface free energy of dust, ${\gamma }_{s-l}$ is interfacial tension between dust-droplet fluid, and ${\gamma }_{l}$ is droplet fluid surface tension. The spreading coefficient for droplet fluid (water) is estimated as *S* = 21.95 mJ/m^2^, which is consistent with the early work^42^. Hence, water droplet spreads over dust surfaces. Droplet fluid infusion over dust takes place in two stages. Firstly, the forces created by the surface tension of droplet fluid and interfacial shear give rise to the formation of a thin layer of fluid over the wetted dust surface. As the infusion time progresses, the liquid spreading rate increases in accordance with Joos’ law^43^. The spreading velocity in the second stage takes the form ${U}_{s}\propto {(3{S}_{s-l}/4\sqrt{{\mu }_{o}{\rho }_{o}})}^{1/2}{t}^{-1/4}$), here *μ*_*o*_ and *ρ*_*o*_ are viscosity and density of droplet fluid^44^. As infusion progress over dust surface, the droplet fluid kinetic energy dissipation occurs under the shearing effect, which can be related to Ohnesorge number ($Oh={\mu }_{o}/\sqrt{{\rho }_{o}a{\gamma }_{l}}$), where *a* represents the characteristic size of the dust particle^16,44^. For droplet fluid (water) infusion over averaged size dust (1.2 μm), Ohnesorge number becomes greater than 1; hence, the kinetic energy of infusing liquid is dissipated by a large amount due to the shearing effect. Figure 9b shows water infusion with time over a dust particle. The dust particle of size more than 20 μm can be covered by infused water within 0.26 s. However, as the dust particle size reduces to average size (1.2 μm), water infusion time reduces to almost 0.016 s. It is worth mentioning that the scaling is used to estimate the infusion time for the average size dust particle (1.2 μm). Since only the dust particles, which are infused by droplet fluid, can be picked by a rolling droplet, the residency of droplet wetted length over the dusty surface is expected to be longer than the total infusion time of droplet fluid covering the particle surface. The contact duration of the wetting length of the rolling droplet over the dusty surface is estimated as 0.025 s for droplet wetting length of 2 mm and droplet translation velocity of 80 mm/s. Consequently, droplet fluid has sufficient time to infuse over the surface of dust during rolling on the dusty surface. In this case, it is expected that droplet fluid picks the dust particles from the dusty surface during its rolling. Figure 10 shows high-speed camera images of droplets rolling over dusty micro-post array surfaces. The dust particles are picked up by rolling droplets from the surface as rolling progress. Figure 11 shows droplet velocity obtained from the experiments due to dusty and clean surfaces for two droplet volumes and various micro-post gap spacings. Droplet velocity attains larger values for the clean surface than that of the dusty surface, which is more pronounced for 50 μm gap spacing of the micro-post arrays. Moreover, as droplet volume increases, droplet velocities on the clean and dusty surfaces become larger than those obtained for small volume droplets. Increasing gravitational force for large droplet masses causes the attainment of high droplet velocity. In addition, increasing micro-post gap spacing lowers droplet wetting length and interfacial contact area of the droplet fluid. This contributes to droplet velocity enhancement during rolling on both dusty and clean surfaces. However, the presence of dust causes droplet deceleration over the surface, i.e. dust particles act like retarding force centers lowering rolling droplet kinetic energy while increasing friction between droplet fluid and the surface^45^. In addition, alkaline salt compounds (NaCl and KCl) in dust can be dissolved in droplet fluid upon picked up during rolling. This changes the surface tension of droplet fluid and modifies slightly pinning force between the rolling droplet and dusty micro-post array surface. The change in surface tension due to the dissolution of salt compounds in droplet fluid does not notably change droplet fluid surface tension^46^. Hence, pinning force enhancement, due to surface tension increase, does not have a considerable effect on droplet velocity. To evaluate droplet kinetic energy dissipation over a dusty surface, the kinetic energy ratio ($\chi$) is introduced in reference to a clean surface. The kinetic energy loss ratio takes the form: $\zeta =\frac{{V}_{d-clean}^{2}-{V}_{d-dust}^{2}}{2glsin\delta }$, where ${V}_{d-clean}$ and ${V}_{d-dusty}$ are droplet velocities on clean and dusty micro-post arrays surfaces, respectively. Figure 12a,b show kinetic energy loss ratio ($\zeta$) with distance for different micro-post gap spacings and two droplet volumes, respectively. The kinetic energy loss ratio increases as the distance increase to 8 mm from the location of initiation of droplet rolling, which is more pronounced for large micro-post spacing. Therefore, the kinetic energy loss ratio becomes larger for large gap spacings. This is because of the magnitude of droplet velocity on clean and dusty micro-post array surfaces. In this case, increasing gap spacing enhances droplet velocity for both clean and dusty surfaces. In addition, the gap spacing has a notable effect on the droplet kinetic energy loss, i.e. for large gap spacing not only the droplet velocity increase, but the kinetic energy ratio also increases. Figure 13 shows optical and scanning electron microscope images of the droplet path for micro-post gap spacing of 25 μm and plain PDMS surface. Droplet picks up dust particles along its path (Fig. 13a,b); however, some small dust residues are observed in gap spacings. The closed examination of dust residues revealed that only a few small size dust particles are present within micro-post gap spacings (Fig. 13c,d). The presence of the dust residues are related to one or all of the followings: (1) droplet fluid cannot completely infuse over dust surfaces while they are not picked up by rolling droplet, and/or (2) some dust particles have sharp edges and they can anchor over the coated micro-post array surfaces, which cannot be picked up by rolling droplet despite that droplet fluid completely infuses over dust surfaces. Nevertheless, the number of dust residues remains small within micro-post gap spacings.

**Table 1:** Elemental composition of dust particles (wt%).

	Size (μm)	Si	Ca	Na	S	Mg	K	Fe	Cl	O
Collected dust	≥ 1.2	11.2	8.1	2.2	1.3	2.4	0.8	1.1	0.4	Balance
Collected dust	< 1.2	10.4	7.4	2.6	2.2	1.4	1.1	1.1	0.9	Balance

## Conclusion

Rolling droplet behavior over hydrophobized micro-post array surfaces is studied and the effect of micro-post array spacing on droplet rolling kinematics is examined for clean and dusty micro-post array surfaces. Experiments are carried out towards monitoring and assessing droplet rolling behavior in terms of rolling and translational velocities over hydrophobized inclined micro-post arrays. Micro-post arrays are replicated over polydimethylsiloxane (PDMS) from initially formed micro-post arrays on silicon wafers via lithography technique. Micro-post array surfaces are hydrophobized through the deposition of functionalized silicon nanoparticles via dip coating. Hydrophobized PDMS micro-post arrays have different gap spacings between the micro-posts. This arrangement enables to examine droplet rolling behavior over micro-post array surfaces having different gap spacings. The flow inside the rolling droplet is numerically simulated and the effect of micro-post gap spacing on the flow structures is predicted. The findings revealed that micro-post array spacing influences the droplet rolling velocity. In this case, micro-post gap spacings act like air cushions at droplet interface lowering droplet wetted diameter and droplet fluid interfacial area. Hence, increasing micro-post spacing results in increased droplet rolling velocity over the surface. The droplet kinetic energy loss increases as the micro-post spacing reduce, which is associated with an increased lateral component of surface tension force and enhancement of interfacial shear resistance. Droplet wobbling during rolling increases the kinetic energy loss, which becomes more apparent for large volume droplets. The complex flow structure is formed inside the rolling droplet fluid and velocity magnitude attains large values for large gap spacings. In the early rolling period, the influence of gap spacing on the flow structure is not notable; however, as the rolling period increases (> 5 ms), the flow structure inside the droplet fluid influences increasing gap spacing. Dust on micro-post array surfaces is picked up by rolling droplets for all micro-post gap spacings considered. However, some dust residues are observed in micro-post spacings; provided that dust residues are not significantly large in amount. The presence of dust contributes to the kinetic energy dissipation of rolling droplets, which becomes notable for large gap spacings. This becomes more pronounced for large volume droplets. The present study explores insight into rolling droplet behavior over hydrophobized clean and dusty micro-post array surfaces and provides details of the influence of gap spacings on droplet dynamic characteristics.

## Supplementary Information

Supplementary Information.
