# Supplementary material for: Water droplet can mitigate dust from hydrophobized micro-post array surfaces
Source: Sci Rep. 2021 Sep 15;11:18361. doi: 10.1038/s41598-021-97847-7 (PMC8443603; doi:10.1038/s41598-021-97847-7)
Supplement: Supplementary file 1 — Supplementary Information. [file 41598_2021_97847_MOESM1_ESM.docx]

**Supporting Information**

**WATER DROPLET CAN MITIGATE DUST FROM HYDROPHOBIZED MICRO-POST ARRAY SURFACES**

Abba Abdulhamid Abubakar^1^ Bekir Sami Yilbas^1,2,3^ Hussain Al-Qahtani^1^ Ghassan Hassan^1,4^ Johnny Ebaika Adukwu^1,4^

1Mechanical Engineering Department, KFUPM, Dhahran 31261, Saudi Arabia.

2Center of Excellence for Renewable Energy, Mechanical Engineering Department, KFUPM, Dhahran 31261, Saudi Arabia.

^3^Senior Researcher at K.A. CARE Energy Research & Innovation Center at Dhahran, Saudi Arabia

^4^Researcher at K.A. CARE Energy Research & Innovation Center at Dhahran, Saudi Arabia

*Correspondence and requests for materials should be addressed to B.S.Y. (Email: bsyilbas@kfupm.edu.sa; Phone: +966 3 860 4481).

**S1: Mesh used in the simulations**

COMSOL multi-physics code^1^ is used to model the dynamics of a rolling droplet on the micro-post array surface incorporating the initial and the boundary conditions presented through Eq. (1) to (26). However, as the droplet rolling length is on a length scale that is far greater than the micro-post pillar and gap sizes, the use of actual micro-post array structures is extremely difficult from numerical point of view. Therefore, the influence of micro-post spacing is incorporated through the frictional force in the numerical scheme. Moreover, time derivatives are discretized based on second-order Euler backward difference scheme and time steps as small as 10^-8^ s are adopted for the numerical solution.

Adopting the level-set approach, the droplet interface is treated implicitly; hence, droplet interface freely moves within the computational grid via a regular mesh of sufficient density and well-calibrated level set parameters (i.e. $\gamma_{r}$ and $\varepsilon_{ls}$). In the present study, the re-initialization parameter ($\gamma_{r}$) is set as 0.6 m/s and the interface thickness ($\varepsilon_{ls}$) is set as 10-15 μm depending on the element edge length. Figure S1 shows the computational grid adopted in the simulations. A mesh consisting of 1,279,003 tetrahedral elements was utilized. Figure S2 shows that the grid-independent solution is obtained is obtained with 1,279,003 tetrahedral elements which have cumulative average element quality of 0.94. The solution shows variation of pressure and velocity magnitude after a total duration of 21 ms along the depth of the 40 µL droplet for a gap spacing of b = 50 µm.. Furthermore, preliminary check shows that mass loss is negligibly small during the droplet rolling simulations down the micro-post array surface.

**Figure S1.** (a) Cross-section of mesh used in simulations, (b) initial location of a droplet in a 3-dimensional domain, (c) meshes used near droplet.

**Figure S2.** (a) centerline along which grid independence test is conducted for 40 µL droplet, inclination angle δ=5°, gap spacing b = 50 µm and t = 0.21 s; (b) velocity magnitude, (c) gauge pressure.

**References**

1. COMSOL Inc. COMSOL Multiphysics. (2017).
